# Extracellular Vesicles from Probiotic and Beneficial *Escherichia coli* Strains Exert Multifaceted Protective Effects Against Rotavirus Infection in Intestinal Epithelial Cells

**DOI:** 10.3390/pharmaceutics18010120

**Published:** 2026-01-18

**Authors:** Cecilia Cordero, Aitor Caballero-Román, Sergio Martínez-Ruiz, Yenifer Olivo-Martínez, Laura Baldoma, Josefa Badia

**Affiliations:** 1 Departament de Bioquímica i Fisiologia, Facultat de Farmàcia i Ciències de l’Alimentació, Universitat de Barcelona, 08028 Barcelona, Spain; corderocecilia16@gmail.com (C.C.); sergio_martinez_ruiz@ub.edu (S.M.-R.); yeni_olivo@hotmail.com (Y.O.-M.); 2 Department de Farmàcia i Tecnologia Farmacèutica, i Fisicoquímica, Facultat de Farmàcia i Ciències de l’Alimentació, Universitat de Barcelona, 08028 Barcelona, Spain; caballeroaitor@ub.edu; 3 Institut de Biomedicina de la Universitat de Barcelona (IBUB), 08028 Barcelona, Spain; 4 Institut de Recerca Sant Joan de Déu (IRSJD), 08950 Barcelona, Spain

**Keywords:** rotavirus, microbiota, probiotics, postbiotics, extracellular vesicles

## Abstract

**Background/Objectives:** Rotavirus remains a major cause of severe acute gastroenteritis in infants worldwide. The suboptimal efficacy of current vaccines underscores the need for alternative microbiome-based interventions, including postbiotics. Extracellular vesicles (EVs) from probiotic and commensal *E. coli* strains have been shown to mitigate diarrhea and enhance immune responses in a suckling-rat model of rotavirus infection. Here, we investigate the regulatory mechanisms activated by EVs in rotavirus-infected enterocytes. **Methods:** Polarized Caco-2 monolayers were used as a model of mature enterocytes. Cells were pre-incubated with EVs from the probiotic *E. coli* Nissle 1917 (EcN) or the commensal EcoR12 strain before rotavirus infection. Intracellular Ca^2+^ concentration, ROS levels, and the expression of immune- and barrier-related genes and proteins were assessed at multiple time points post-infection. **Results:** EVs from both strains exerted broad protective effects against rotavirus-induced cellular dysregulation, with several responses being strain-specific. EVs interfered with viral replication by counteracting host cellular processes essential for rotavirus propagation. Specifically, EV treatment significantly reduced rotavirus-induced intracellular Ca^2+^ mobilization, ROS production, and COX-2 expression. In addition, both EV types reduced virus-induced mucin secretion and preserved tight junction organization, thereby limiting viral access to basolateral coreceptors. Additionally, EVs enhanced innate antiviral defenses via distinct, strain-dependent pathways: EcN EVs amplified IL-8-mediated responses, whereas EcoR12 EVs preserved the expression of interferon-related signaling genes. **Conclusions:** EVs from EcN and EcoR12 act through multiple complementary mechanisms to restrict rotavirus replication, spread, and immune evasion. These findings support their potential as effective postbiotic candidates for preventing or treating rotavirus infection.

## 1. Introduction

Rotavirus (RV) infection remains a leading cause of severe acute gastroenteritis in infants worldwide, contributing substantially to morbidity and mortality, particularly in low-income regions [1]. RV is a non-enveloped, double-stranded RNA virus of the *Reoviridae* family that primarily infects mature enterocytes at the villus tips of the small intestine [2]. Infection disturbs calcium signaling, epithelial barrier integrity, electrolyte balance and oxidative stress responses, collectively leading to diarrhea and mucosal damage [3]. Several viral proteins mediate these pathogenic effects. The nonstructural protein 4 (NSP4) functions as a viroporin, inducing Ca^2+^ efflux from the endoplasmic reticulum and thereby disrupting cellular Ca^2+^ homeostasis [4]. Elevated cytosolic Ca^2+^ levels promote mitochondrial dysfunction, redox imbalance and an intracellular environment favorable for viral replication. In turn, excessive Ca^2+^ and reactive oxygen species (ROS) activate downstream signaling pathways that drive inflammation, increase intestinal permeability and exacerbate diarrhea [5]. Additionally, the capsid spike protein VP4 contributes to viral entry through its trypsin-mediated cleavage product, the VP8* peptide, which disrupts apical tight junctions (TJs). This disruption exposes basolateral integrin-type receptors, facilitating viral attachment and internalization [6].

The first line of defense against RV infection is the mucus layer that covers the intestinal epithelium. This layer is rich in secreted mucins, which are highly glycosylated molecules containing complex O-glycans capable of binding RV particles and limiting their access to enterocytes [7,8,9]. To bypass this protective barrier, RV can modify mucin-glycosylation patterns, thereby reducing their neutralizing capacity [10], and induce early alterations in the ileal microbiota that favor the expansion of mucin-degrading bacteria such as *Akkermansia* [11].

Following pathogen invasion, the host immune system is rapidly activated to combat infection. The innate immune response to viral infection is characterized by the rapid induction of type I interferons (IFN-α and IFN-β). This response is initiated upon recognition of viral RNA by host immune receptors. RV double-stranded RNA is primarily detected by toll-like receptor (TLR)-3 and retinoic acid-inducible gene 1 (RIG-I). Their activation triggers signaling cascades that activate transcription factors such as nuclear factor kappa B (NF-κB) and interferon regulatory factor 3 (IRF3), which drive IFN expression [12,13]. Secreted type I IFNs then bind to specific cell surface receptors, leading to the activation of the signal transducers and activation of transcription (STAT)-1 and 2, and the subsequent induction of interferon-stimulated genes (ISG), whose products mediate antiviral defense and viral clearance [14,15]. Among these, ISG15 plays a key antiviral role through covalent conjugation to host and viral proteins, thereby inhibiting viral replication and modulating immune and repair pathways [16]. To counteract this response, RV employs several immune evasion mechanisms, including suppression of RIG-I expression, degradation of IRF3, and inhibition of STAT1/STAT2 nuclear translocation [12,17]. The magnitude of IFN suppression varies among RV strains and host cell types [18]. Moreover, NF-κB activation in infected enterocytes promotes the expression of proinflammatory and chemoattractant mediators such as interleukin (IL)-8, which recruit neutrophils and macrophages to the infection site [19].

Mounting evidence indicates that the gut microbiota profoundly shapes host responses to intestinal pathogens. In this context, microbiome-targeted interventions, including probiotics, prebiotics, and nutritional strategies, are emerging as promising approaches to enhance immune function, reduce infection risk, and potentially improve vaccine efficacy [20,21,22]. Probiotic bacteria such as *Bifidobacterium*, *Lactobacillus*, and *Escherichia coli* strains have been investigated for their ability to prevent or mitigate RV infection by strengthening mucosal immunity and supporting intestinal homeostasis in experimental models, particularly those involving neonatal animals [23,24,25,26,27,28]. Nevertheless, therapies based on live bacteria face efficacy and safety limitations, particularly in newborns and immunocompromised individuals. As a result, current biotic strategies increasingly focus on postbiotics [29,30]. Postbiotics are non-viable microbial components or products—such as cell wall fragments, lysates, secreted factors, and metabolites—that, when administered in adequate amounts, can confer health benefits comparable to those of live probiotics [31].

Among postbiotics, extracellular vesicles (EVs) produced by beneficial gut bacteria are emerging as promising tools for the prevention and management of a wide range of human diseases, including infectious disorders [32,33,34]. Bacterial EVs are typically spherical, lipid-bilayer nanostructures ranging from 20 to 300 nm in diameter. They are natural carriers of bacterial molecules, including proteins, lipids, enzymes, metabolites and nucleic acids, and function as long-distance delivery systems for bioactive cargo. Analogous to eukaryotic exosomes, bacterial EVs are key mediators of intercellular communication [35]. They interact with target cells and can be internalized through multiple endocytic pathways, facilitating targeted cargo delivery [36]. Microbiota-derived EVs are now recognized as pivotal mediators of microbiota-host interactions. Within the gut, they can traverse the mucus layer, interact with epithelial and immune cells, cross host epithelial and endothelial barriers and modulate host signaling pathways and functions. Through their molecular payload, microbiota EVs exert multifaceted immunomodulatory and barrier-protective effects, mediating the effects of microbiota and probiotics strains on host physiology and health [37].

Beyond studies in experimental models of inflammatory, metabolic and neurological diseases, preclinical assays aimed at evaluating the efficacy of microbiota or probiotic-derived EVs in RV infection models remain largely unexplored. Recent work from our group has shown the benefits of interventions involving EVs from the probiotic *Escherichia coli* Nissle 1917 (EcN) and the commensal EcoR12 in a suckling-rat model of RV infection. Both treatments mitigated diarrhea and promoted humoral immunity, as evidenced by increased serum immunoglobulin levels, and strengthened cellular antiviral defenses, including enhanced splenic natural killer cells, cytotoxic T lymphocytes, and T-cell populations expressing the T cell receptor (TCR) γδ^+^. Some effects, however, differed between strains. EcoR12 EVs stimulated intestinal *CD68*, *TLR2*, and *IL12* expression, whereas EcN-derived EVs supported intestinal development by improving epithelial barrier features and by increasing absorptive capacity, reflected in greater villus height [38]. These findings provided the first evidence that probiotic- and microbiota-derived EVs constitute a safe, bacteria-free postbiotic strategy to mitigate neonatal RV disease and may also serve as adjuvants to enhance RV vaccine efficacy. Nevertheless, their translation into dietary supplements or therapeutic formulations requires a comprehensive understanding of their molecular mechanisms of action. Beyond the immunomodulatory effects observed in suckling rats, the specific molecular alterations triggered by EcN- or EcoR12-derived EVs in enterocytes during RV infection, particularly those that may inhibit viral replication or counteract viral immune-evasion strategies, have yet to be elucidated.

The present study aimed to elucidate the mechanisms underlying the protective actions of EcN- and EcoR12-derived EVs against RV-induced disturbances in polarized Caco-2 cell monolayers, used as a model of mature enterocytes. Particular attention was given to pathways related to calcium homeostasis, oxidative stress, epithelial barrier integrity, and antiviral immune responses. Defining these mechanisms will advance our understanding of microbiota-host interactions during viral infection and may facilitate the development of safe and effective postbiotic-based interventions for viral gastroenteritis.

## 2. Materials and Methods

### 2.1. Bacterial Strains and Isolation of Extracellular Vesicles (EVs)

EVs were isolated from two intestinal *E. coli* strains, the probiotic EcN (Ardeypharm GmbH, Herdecke, Germany) and the commensal EcoR12 strain from the ECOR reference collection [39]. Both strains were cultured in Luria–Bertani (LB) broth under standard growth conditions.

EV isolation was performed as previously described [40] with minor modifications. Briefly, bacterial cultures were centrifuged at 10,000× *g* for 20 min at 4 °C to remove cells, and the resulting supernatants were filtered through 0.22 µm pore-size membranes (Merck Millipore, Burlington, MA, USA) to eliminate residual bacteria. The cell-free filtrates were concentrated using Centricon Plus-70 centrifugal devices with a 100 kDa molecular weight cutoff (Merck Millipore, MA, USA). EVs were then collected by ultracentrifugation at 150,000× *g* for 1 h at 4 °C using a Type 90 Ti rotor (Beckman Coulter Inc., Brea, CA, USA), washed twice with phosphate-buffered saline (PBS), and resuspended in PBS. The sterility of the EV preparations was confirmed by plating on LB agar, and EV protein concentration was determined using the Pierce BCA Protein Assay Kit (Thermo Fisher Scientific, Barcelona, Spain). Vesicle quantification was additionally performed using the lipophilic fluorescent probe FM4-64 (Thermo Fisher Scientific, Barcelona, Spain), which incorporates into vesicle membranes. This approach was used to confirm that comparable quantities of EcN-derived EVs and EcoR12-derived EVs were applied throughout the experimental procedures [38]. For the FM4-64 assay, serial dilutions of EV stock solutions (40 μg/mL) were incubated with FM4-64 (5 μg/mL in PBS) for 10 min at room temperature. Controls containing only EVs or only the fluorescent dye were included. Fluorescence was recorded using a Varioskan Lux multiplate reader (Thermo Fisher Scientific, Barcelona, Spain) following excitation at 515 nm and emission detection at 640 nm. EV preparations adjusted to the same protein concentration (µg protein/mL) exhibited comparable fluorescence signals. Finally, EV samples were aliquoted and stored at −20 °C until further use. Each aliquot was used only once to avoid repeated freeze–thaw cycles.

The native morphology, size distribution and structural integrity of EcN- and EcoR12-derived EVs had been previously examined using cryo–transmission electron microscopy [41].

### 2.2. Rotavirus Strain

The simian SA11 RV strain was provided by the Enteric Virus Laboratory of the University of Barcelona. The viruses were propagated in fetal African green monkey kidney (MA-104) cells and titrated as plaque-forming units (PFU).

### 2.3. Cell Culture and Infection Conditions

Human colonic epithelial Caco-2 cells (ATCC HTB-37) and human goblet LS174T cells (ATCC CL-188) were maintained in Dulbecco’s Modified Eagle Medium (DMEM) and Roswell Park Memorial Institute (RPMI)-1640 medium, respectively. Both culture media were supplemented with 10% fetal bovine serum (FBS), 25 mM HEPES, 1% non-essential amino acids, and 1× penicillin–streptomycin (Corning, Fisher Scientific Inc., Barcelona, Spain, Cat. No. 30-002-CI). Cells were cultured at 37 °C in a humidified incubator with 5% CO_2_. Cultures were passaged upon reaching 80–90% confluence for subsequent experiments.

Caco-2 cells were seeded at a density of 2 × 10^5^ cells/mL and cultured until full differentiation (15 days post-confluence), with medium replaced every two days. For infection experiments, differentiated monolayers were pre-incubated with EcN or EcoR12 EVs for 16 h in serum-free medium. Control cells were maintained in serum-free medium under the same conditions. Prior to infection, cells were washed to remove non-internalized EVs. Rotavirus was activated by incubation with porcine pancreatic trypsin (Sigma-Aldrich, Chemical Co., St. Louis, MO, USA) at a final concentration of 10 μg/mL in Hanks’ Balanced Salt Solution (Sigma-Aldrich, Chemical Co., St. Louis, MO, USA) for 1 h at 37 °C. The activated virus was then diluted in serum-free culture medium. Infection was performed at 37 °C for 1 h at a multiplicity of infection (MOI) of 4. Following infection, cells were washed to remove unbound viral particles and incubated in serum-free DMEM for the indicated times post-infection, according to the experimental design.

The same infection protocol was applied to confluent LS174T cells, using the appropriate growth medium and culture conditions. In all cases, culture supernatants and cell pellets were collected for analysis.

### 2.4. Cell Viability and Cytotoxicity Assays

Cell viability was evaluated at 24 h post-infection (hpi) in Caco-2 cells exposed to different rotavirus doses (MOI 2–20), following the infection protocol described above. Viable and non-viable cells were distinguished using the Trypan Blue exclusion assay, and cell counts were performed with a Countess Automated Cell Counter (Invitrogen, Carlsbad, CA, USA). Cytotoxic effects were assessed using the LDH-Cytotoxicity Assay Kit II (Cat. No. ab65393, Abcam, Cambridge, UK), according to the manufacturer’s instructions. This assay quantifies lactate dehydrogenase (LDH) release as an indicator of cell membrane damage.

### 2.5. Quantitative Reverse Transcription-Polymerase Chain Reaction (RT-qPCR)

Both rotavirus VP6 RNA and mRNA levels of selected cellular genes were measured using RT-qPCR. Total RNA was isolated from Caco-2 and LS174T cells with the miRNeasy Mini Kit (Qiagen, Crawley, UK) following the manufacturer’s instructions. RNA quality and concentration were assessed by the ratio of absorbance at 260/280 nm using a NanoDrop TM-2000 spectrophotometer (Thermo Fisher Scientific, Barcelona, Spain). All samples had 260/280 ratio values between 1.9–2.0. Complementary DNA (cDNA) synthesis was performed in a 20 µL reaction using the High-Capacity cDNA Reverse Transcription Kit (Applied Biosystems, Foster City, CA, USA).

Quantitative PCR was conducted on a QuantstudioTM^3^ real-time PCR system (Applied Biosystems, Foster City, CA, USA) utilizing SYBR^®^ Green PCR Master Mix (Applied Biosystems) and gene-specific primers listed in Table S1. *GAPDH* was used as the internal reference gene. The thermal cycling program included an initial denaturation step at 95 °C for 10 min, followed by 40 cycles of 95 °C for 15 s and 60 °C for 1 min. Negative control reactions lacking RNA were included to ensure specificity. Relative RNA expression levels were calculated using the 2^−ΔΔCt^ formula, and results were presented as fold changes relative to the appropriate control group (untreated cells or rotavirus-infected cells, depending on the experiment).

### 2.6. Intracellular Calcium Quantification

Intracellular calcium levels were measured using the fluorescent probe Fluo-4 AM (Molecular Probes- F14201, Thermo Fisher Scientific, Barcelona, Spain). Caco-2 cells were cultured in 35 mm Ibidi plates in phenol red-free DMEM until post-confluency. Cells were then incubated with EVs from EcN or EcoR12 strains at concentrations of 5 and 50 µg/mL in serum-free DMEM for 16 h prior to rotavirus infection (MOI 4) as described above. After 1 h of infection, cells were washed and maintained in serum-free DMEM. At 8 hpi, cells were washed three times with Hank’s Balanced Salt Solution and incubated in the dark with Fluo-4 AM for 40 min at 37 °C, followed by washing and processing according to the manufacturer’s instructions. The time point was selected based on previous reports showing that rotavirus replication induces an increase in intracellular calcium starting at 7 hpi [42].

Fluorescence was measured using a THUNDER fluorescence microscope (Leica, Wetzlar, Germany) with excitation/emission wavelengths of 494/506 nm on the Unit from Scientific and Technological Centers (CCiTUB), Universitat de Barcelona. For quantification, five fields per experimental condition were analyzed, with three independent replicates (n = 3).

### 2.7. Quantification of Reactive Oxygen Species (ROS)

Intracellular ROS levels were measured using the cell-permeable oxidation-sensitive dye 2′,7′-dichlorofluorescin diacetate (DCFDA) (Sigma-Aldrich, Chemical Co., St. Louis, MO, USA).

Caco-2 cells were seeded in 96-Well Optical BTM Plt Polymerbase Black W/lid, plates (Thermo Fisher Scientific, Barcelona, Spain) at a density of 1 × 10^5^ cells/cm^2^ and cultured in phenol red-free DMEM. Prior to rotavirus infection, cells were incubated for 16 h in serum-free DMEM in the absence or presence of EcN or EcoR12 EVs (0.5 µg/mL). Cells were then washed and incubated with 25 µM DCFDA at 37 °C for 45 min in the dark. Fluorescence was immediately measured (excitation/emission: 485/530 nm) using a Varioskan^TM^ Lux plate reader (Thermo Fisher Scientific, Barcelona, Spain) to establish the baseline (time zero). Rotavirus was subsequently added (MOI of 4) to the appropriate wells. Uninfected cells served as negative controls. Plates were incubated in the dark at 37 °C in a CO_2_ incubator. Fluorescence was recorded at 30, 60, and 120 min post-infection. The results were presented as the percentage of fluorescence increase compared to non-infected control cells.

### 2.8. Enzyme-Linked Immunosorbent Assay (ELISA)

The secreted levels of IL-8 were quantified using ELISA sets (BD Biosciences, San Jose, CA, USA) according to the manufacturer’s instructions. For this assay, Caco-2 cell monolayers were infected with rotavirus (MOI 4) as described in Section 2.3. At the indicated post-infection times, culture supernatants were collected, centrifuged at 10,000× *g* for 20 min at 4 °C and stored at −80 °C until analysis.

### 2.9. Western Blot

Time-course RV infection was evaluated by detecting the viral structural protein VP6 at various time points post-infection using immunoblotting. Cells were washed with ice-cold PBS, resuspended in RIPA lysis buffer (Sigma-Aldrich), and vortexed for 30 s. Lysates were centrifuged at 15,000× *g* for 15 min at 4 °C, and the resulting supernatants were collected and stored at −80 °C until analysis. Equal amounts of protein were separated by 10% SDS-PAGE and transferred onto a Hybond-P polyvinylidene difluoride membrane by using a Bio-Rad Mini Trans-blot system (Alcobendas, Madrid, Spain). Membranes were blocked for 1 h at room temperature with PBS containing 0.05% Tween-20 and 5% skimmed milk (blocking solution), followed by overnight incubation at 4 °C with an anti-VP6 primary antibody (sc101363, 1:2000 dilution, Santa Cruz Biotechnology, Dallas, TX, USA). After washing, membranes were incubated with horseradish peroxidase-conjugated rabbit anti-mouse IgG secondary antibody (sc-516102; 1:5000 dilution; Santa Cruz Biotechnology). GAPDH was used as a loading control. Protein bands were visualized using the ECL Plus detection system (Amersham Pharmacia Biotech, Piscataway, NJ, USA), and densitometric quantification was performed using Image Studio^TM^ Lite software v5.2.5 (LI-COR, Biosciences, Lincoln, NB, USA). Data were expressed as the relative intensity (%) compared to VP6 levels in Caco-2 cells at 24 hpi.

### 2.10. Immunofluorescence Labeling and Confocal Microscopy Analysis

Caco-2 cells were seeded at a density of 1 × 10^4^ cells/mL in an 8-well chamber slide (µ-Slide 8 well Glass bottom, Ibidi GmbH, Gräfelfing, Germany) and cultured for 10 days. Cells were then incubated with EVs (50 µg/mL) in serum-free medium and infected with RV SA11 as described in Section 2.3 (1 h, MOI 4). At the indicated post-infection times, cells were washed in glycine-PBS buffer (pH 7.0), fixed with 4% paraformaldehyde and permeabilized with 0.05% saponin. Blocking was performed using glycine-PBS buffer containing 1% bovine serum albumin as previously described [43].

The TJ proteins zona occludens (ZO)-1, occludin, claudin-1, and claudin-2 were detected using specific primary antibodies: mouse anti-ZO-1 (ZO-1-1A12, 5 µg/mL, Invitrogen), rabbit anti-occludin (0.5 µg/mL, Invitrogen), rabbit anti-claudin-1 (EPRR18871, 5 µg/mL, Abcam), and rabbit anti-claudin-2 (5 µg/mL, Abcam). Cells were incubated with the primary antibodies for 16 h at 4 °C, followed by a 3 h incubation with the appropriate secondary antibody, Alexa Fluor 488-conjugated F(ab’)_2_ goat anti-mouse IgG (H+L) (5 µg/mL, Invitrogen) or Alexa Fluor 488-conjugated F(ab’)_2_ goat anti-rabbit IgG (H+L) (5 µg/mL, Invitrogen). Nuclei were counterstained with DAPI (0.125 µg/mL, Sigma-Aldrich, St. Louis, MO, USA) for 20 min at room temperature.

Cell imaging was performed using a Zeiss LSM880 confocal microscope (Carl Zeiss Iberia S.L., Jena, Germany) equipped with a 63×/1.4 objective lens, an Argon laser, and a 405 nm laser diode. The 488 nm emission line from the Argon laser was employed to excite Alexa Fluor 488, while the 405 nm diode was utilized to excite DAPI fluorescence. Image stacks were acquired at a voxel resolution of 0.13 µm × 0.13 µm × 0.37 µm along the x, y, and z axes, respectively. Subsequent image processing and quantitative analyses were carried out using Fiji software v1.53t. Images were visualized using the Fire look-up table (LUT) to represent fluorescence intensity differences (low intensity = dark colors; high intensity = bright colors). This LUT was applied in Fiji/ImageJv1.53t for all displayed micrographs.

### 2.11. Mucin Expulsion Assay

The assay was conducted using the mucin-secreting LS174T cell line. Cells were grown to confluence in 6-well plates, and pre-incubated with EVs in serum-free RPMI medium for 16 h. Following incubation, cells were washed with serum-free medium and subsequently infected with rotavirus strain SA11 for 1 h (MOI 4). After infection, cells were washed and maintained at 37 °C for 12 h and 24 h. At each time point, culture supernatants were collected for mucin quantification using Alcian Blue staining as described elsewhere [11]. Briefly, supernatants were mixed with an equal volume of 1% Alcian Blue solution prepared in 3% glacial acetic acid and incubated for 2 h at room temperature. Mucin complexes were pelleted by centrifugation at 12,000× *g* for 10 min and washed three times with PBS to remove unbound dye. The resulting pellets were resuspended in PBS containing 10% SDS and sonicated to release bound Alcian Blue. Absorbance was measured at 620 nm using Varioscan^TM^ Lux microplate reader (Thermo Fisher Scientific, Barcelona, Spain). Mucin concentrations were determined using a standard curve generated with porcine stomach mucus (M2378, Sigma-Aldrich, St. Louis, MO, USA).

### 2.12. Statistical Analysis

Data were obtained from at least three independent biological replicates (n = 3), each performed in triplicate as technical replicates. Statistical analyses and graph preparation were carried out using GraphPad Prism version 9.4.1 (GraphPad Software, La Jolla, CA, USA). Data were tested for normal distribution using the Shapiro–Wilk test. Results are expressed as mean ± standard error of the mean (SEM). Comparisons among groups were performed using one-way analysis of variance (ANOVA) followed by Tukey’s multiple comparison test. Differences were considered statistically significant at *p* < 0.05.

## 3. Results

### 3.1. EcN- and EcoR12-Derived EVs Reduce Rotavirus Infection in Caco-2 Cell Monolayers

**Figure 1:**
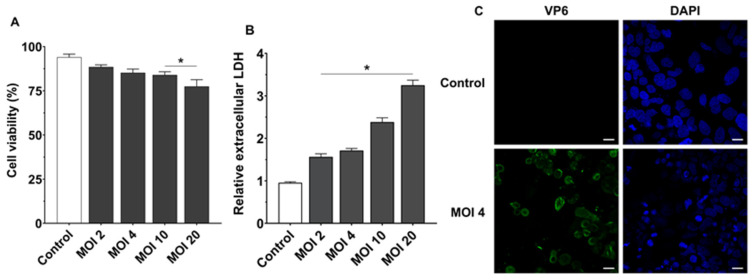
Dose-dependent cytotoxicity of RV strain SA11 in Caco-2 cells. Caco-2 cell monolayers were challenged with RV strain SA11 at the indicated MOI. Following a 1 h infection period, cells were washed and incubated in serum-free medium for 24 h. (**A**) Cell viability was determined using the trypan blue exclusion assay. (**B**) RV-induced cytotoxicity was quantified by measuring LDH release in culture supernatants. Data are presented as relative levels of released LDH compared to untreated control cells (set at 1). In both panels, data are expressed as mean ± SEM from three biological replicates. Statistical differences were assessed by one-way ANOVA, followed by post hoc Tukey’s. * *p* ≤ 0.05 vs. untreated control cells. (**C**) Immunofluorescence detection of VP6 in RV-infected Caco-2 cells (MOI 4) at 24 hpi. VP6 was detected by immunofluorescence staining (green), and nuclei were counterstained with DAPI (blue). Representative confocal maximum projection images are shown. Scale bar, 20 µm.

Prior to assessing the effects of EVs on rotavirus infection and replication in Caco-2 cell monolayers, the infection model conditions were established. A 1 h infection protocol was selected, followed by washing to remove non-internalized viral particles and subsequent incubation in serum-free DMEM. This protocol was adapted from published studies [44,45]. Using this experimental setup, the effect of increasing rotavirus doses (MOI 2–20) on cell viability was evaluated by trypan blue exclusion assay at 24 hpi (Figure 1A). The results showed that higher viral doses (MOI 10–20) caused a significant reduction in cell viability compared to controls, whereas doses up to an MOI of 4 maintained cell viability comparable to that of uninfected cells.

In addition, cytotoxicity induced by RV was evaluated as a function of the applied MOI using the LDH enzymatic activity assay in the culture supernatant (Figure 1B). This enzyme is only released into the medium when the cell membrane is compromised; therefore, extracellular LDH activity serves as an indicator of cell damage. The results showed a clear correlation between the levels of secreted LDH and the RV dose. At MOI of 4 or lower, extracellular LDH levels were close to control values and did not exceed a twofold increase (Figure 1B).

Based on these findings, the dose corresponding to a MOI of 4 was selected for subsequent experiments. Under these infection conditions, immunofluorescence analysis by confocal microscopy of intracellular VP6 demonstrated that infection was effective, as viral particles internalized after 1 h of incubation were able to replicate and express the VP6 protein (Figure 1C).

**Figure 2:**
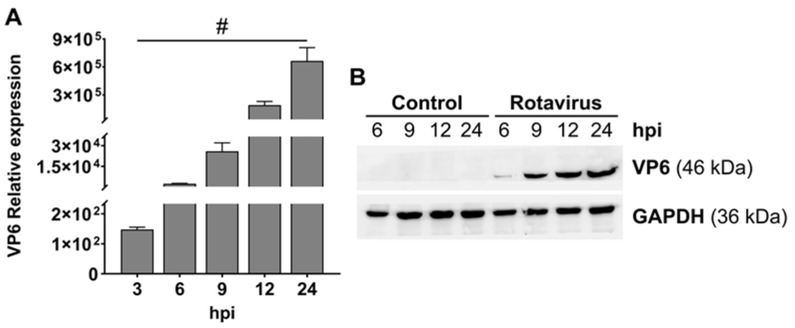
Relative expression of viral VP6 RNA and protein in RV-infected Caco-2 cells. Cell were infected with RV SA11 strain (MOI 4) for 1 h (time 0). (**A**) VP6 RNA levels were quantified by RT-qPCR and normalized to GAPDH expression. Results are expressed as fold change relative to VP6 RNA levels at time 0. Statistical significance: # *p* ≤ 0.05 vs. 0 hpi. (**B**) VP6 protein levels were analyzed in cell extracts by Western blot (25 μg protein/well). GAPDH was used as an internal loading control. (**A**) Representative Western blot images are shown.

Replication of RV was evaluated by quantifying *VP6* RNA levels in infected Caco-2 cell monolayers at various time points using RT-qPCR. *VP6* RNA was detectable as early as 3 hpi and increased progressively up to 24 hpi (Figure 2A). Consistently, Western blot analysis demonstrated a time-dependent accumulation of VP6 protein in RV-infected Caco-2 cells, with expression first detected at 6 hpi and reaching maximal levels at 24 hpi (Figure 2B).

**Figure 3:**
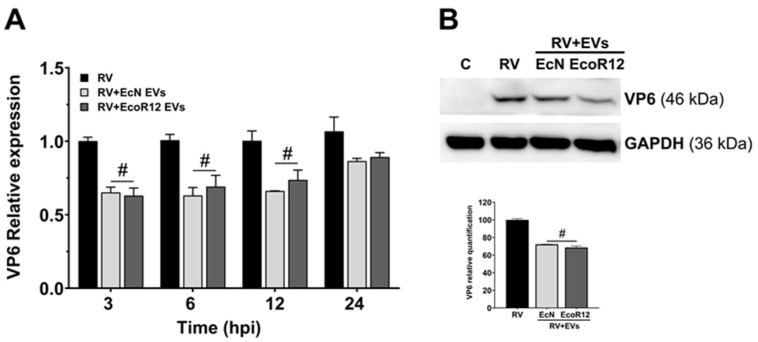
EcN and EcoR12 EVs reduce VP6 RNA and protein levels in Caco-2 cells. Prior to RV infection (MOI 4, 1 h), cells were incubated with EVs (5 µg/mL) in serum-free medium for 16 h. At the indicated times post-infection, cells were processed for RNA or protein extraction. (**A**) VP6 RNA levels were quantified by RT-qPCR and normalized to GAPDH expression. Results are expressed as fold change relative to VP6 RNA levels in RV-infected cells at the same incubation times. (**B**) VP6 protein levels were analyzed in cell extracts by Western blot at 24 hpi. GAPDH was used as an internal loading control. Representative Western blot images from three independent experiments are shown in the upper panel, and the densitometric quantification is shown below. Normalized values from RV-infected cells were set as 100%. Statistical significance: # *p* ≤ 0.05 compared to RV-infected control cells.

To evaluate the effect of microbiota-derived EVs on RV replication, differentiated Caco-2 cells were pre-incubated with EVs derived from the probiotic EcN or the commensal strain EcoR12 (5 μg/mL) for 16 h prior to RV infection. Parallel cultures without EV treatment served as controls. *VP6* RNA levels were quantified by RT-qPCR at various time points post-infection (Figure 3A). In EV-treated cells, regardless of the strain of origin, *VP6* RNA levels were significantly lower than in untreated controls. These differences were detectable as early as 3 hpi, remained statistically significant up to 12 hpi, and followed the same trend at later time points (24 hpi). Consistent with the RNA data, VP6 protein expression was also reduced in EV-pretreated cells compared with control RV-infected cells (Figure 3B). Together, these findings indicate that EVs derived from intestinal *E. coli* microbiota strains can inhibit or interfere with the replication and infectivity of rotavirus in mature enterocytes at early stages of the infectious process.

### 3.2. EcN and EcoR12 EVs Interfere with Key Processes Required for RV Replication and Virion Assembly

It is well established that RV manipulates multiple host cellular processes to create a favorable environment for its replication and propagation. In this context, Ca^2+^ mobilization, oxidative stress and cyclooxygenase (COX)-2 activation play pivotal roles in RV replication and assembly [3,5,46,47,48].

**Figure 4:**
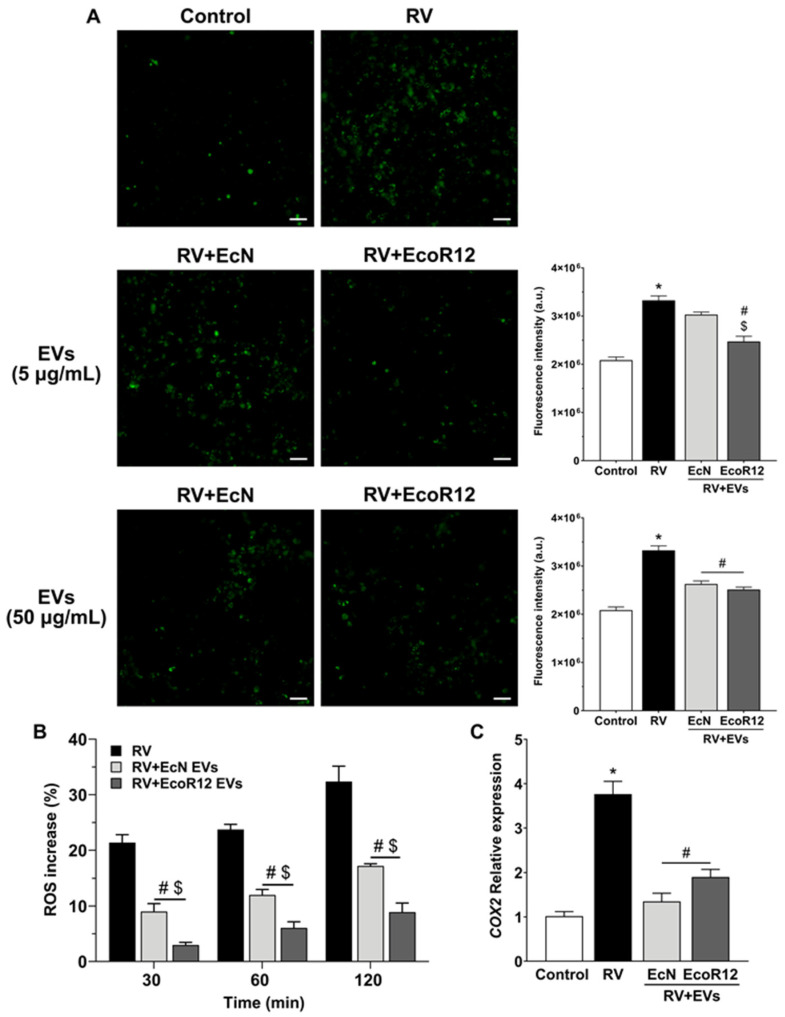
EcN and EcoR12 EVs reversed the RV-induced activation of Ca^2+^ mobilization, ROS production and *COX2* expression. (**A**) Intracellular Ca^2+^ levels were determined by fluorescence microscopy using the Fluo-4 AM probe at 8 hpi (excitation/emission wavelengths: 494/506 nm). Representative images are shown on the left. Scale bar, 50 µm. For quantification, fluorescence was analyzed in 5 fields for each experimental condition and replicate (n = 3). Quantification results are shown in the graphs on the right. (**B**) Intracellular ROS levels were measured using the DCFH-DA probe at the indicated time points in RV-infected cells pre-treated with EVs (0.5 µg/mL) derived from the indicated strains. Data are expressed as the percentage increase in fluorescence relative to untreated control cells at the corresponding time points. (**C**) *COX2* mRNA expression levels were quantified by RT-qPCR at 24 hpi in RV-infected cells pre-treated with EVs (5 µg/mL) isolated from the indicated strains. Relative expression was calculated using *GAPDH* as the internal normalization control. In all panels, results are expressed as mean ± SEM from three biological replicates. Statistical significance: * *p* ≤ 0.05 compared to untreated control cells; # *p* ≤ 0.05 compared to RV-infected cells; ^$^ *p* ≤ 0.05 between EV-treatments.

RV induces an elevation of cytosolic Ca^2+^ concentrations in enterocytes, primarily resulting from enhanced Ca^2+^ release from the endoplasmic reticulum and increased Ca^2+^ influx through host membrane channels. The sustained rise in intracellular Ca^2+^ levels activates autophagy, a process essential for efficient RV replication, as well as for other pathogenic mechanisms [46]. In this study, intracellular Ca^2+^ levels were quantified by measuring the fluorescence intensity emitted by the Fluo-4 AM probe at 8 hpi in Caco-2 cells infected with RV SA11 (MOI 4). Parallel analyses were performed in cells preincubated for 16 h with EVs from EcN or EcoR12 at concentrations of 5 μg/mL or 50 μg/mL (Figure 4A).

RV infection induced a significant increase in intracellular Ca^2+^ levels compared with uninfected control cells. Importantly, preincubation with EVs from the indicated intestinal *E. coli* strains markedly attenuated the infection-associated rise in calcium levels. No differences were observed between strains at the higher EV dose (50 μg/mL). However, at 5 μg/mL, EVs from commensal EcoR12 exhibited a more pronounced preventive effect than those from strain EcN (Figure 4A). The EV-mediated reduction in intracellular calcium levels is consistent with the decreased viral replication inferred from VP6 RNA levels.

Although elevated ROS levels induced by viral infection activate innate antiviral responses, many studies have shown that ROS production can actually facilitate virus replication and survival [49,50,51]. In this context, we aimed to evaluate the impact of EcN- and EcoR12-derived EVs on ROS production in Caco-2 cells infected with RV SA11 preincubated with EVs (0.5 μg/mL) for 16 h prior to infection. ROS levels were analyzed during the first 2 h of infection using the fluorescent probe DCFDA, as described in the Methods Section 2.7 (Figure 4B). In RV-infected Caco-2 cells, ROS levels increased progressively over time compared with uninfected controls, showing increases of approximately 20% at 30 min post-infection, 25% after 1 h, and over 30% at 2 h. In contrast, cells pretreated with EVs derived from EcN or EcoR12 exhibited a significantly attenuated ROS response at all time points, with the strongest inhibitory effect observed in cells exposed to EVs from the commensal EcoR12. These findings demonstrate that EVs from both strains can effectively reduce the oxidative stress induced by RV during the early phase of infection in intestinal epithelial cells.

The inflammatory enzyme COX-2 and its reaction product, prostaglandin E_2_ (PGE_2_), have been described as key mediators of RV infection at a post-binding stage, activating pathways required for virus replication and protein synthesis [52]. Consistent with these findings, *COX2* expression was markedly upregulated in RV-infected cells, with mRNA levels approximately four-fold higher than those of control cells. Pretreatment with EVs derived from EcN or EcoR12 effectively prevented *COX2* overexpression, preserving expression levels close to those of control cells (Figure 4C).

### 3.3. EVs from EcN and EcoR12 Differentially Enhance Antiviral Innate Immunity in Epithelial Cells

The innate immune response is the first line of defense against an invading pathogen. The antiviral innate immune response is characterized by the induction of type I IFN and the production of pro-inflammatory cytokines and neutrophil/macrophage-recruiting mediators. However, as part of their infection strategy, viruses have evolved diverse mechanisms to subvert the host antiviral response and establish infection [53].

**Figure 5:**
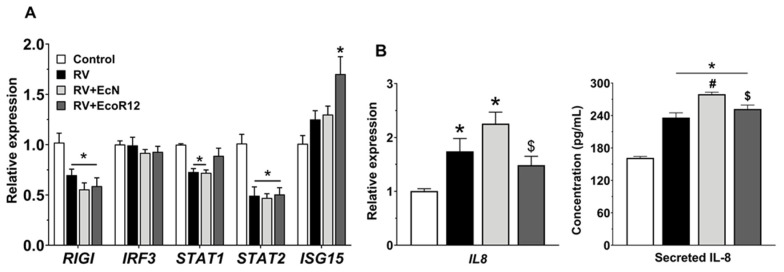
Modulation of the antiviral innate immune response by EcN- or EcoR12-derived EVs in RV-infected Caco-2 cells. Prior to RV infection (MOI 4, 1 h), cells were incubated with EVs (5 µg/mL). At 24 hpi, cells were processed for RNA isolation. (**A**) The transcription levels of genes of the interferon pathway were quantified by RT-qPCR using *GAPDH* as the reference gene. (**B**) Expression of the pro-inflammatory cytokine IL-8 was analyzed at the mRNA level by RT-qPCR (left graph) and quantifying the secreted protein by ELISA (right graph). Relative mRNA levels in infected and treated cells were calculated with respect to the control group (expression value set to 1). In all panels, results are expressed as mean ± SEM from three biological experiments. Statistical significance: * *p* ≤ 0.05 compared to untreated control cells; # *p* ≤ 0.05 compared to RV-infected cells; ^$^ *p* ≤ 0.05 between EV-treatments. *RIG-I*, retinoic acid-inducible gene 1; *IRF3*, interferon regulatory factor 3; *STAT1* and *STAT2*, signal transducer and activator of transcription 1 and 2; *ISG15,* interferon-stimulated gene 15; *IL8*, interleukin-8.

To evaluate whether EVs from EcN and EcoR12 could modulate the type I IFN-mediated antiviral response, we analyzed the expression of genes involved in this pathway in Caco-2 cells following the established infection model at 24 hpi. The analysis included genes encoding the cytosolic pathogen recognition receptor RIG-I, the transcription factors IRF3, STAT1 and STAT2, and the antiviral effector protein ISG15. Infection with RV SA11 significantly reduced mRNA levels of *RIG-I, STAT1* and *STAT2*. Pre-incubation with EcN or EcoR12 EVs did not prevent the virus-induced downregulation of *RIG-I* or *STAT2*. However, EVs from the commensal EcoR12 were able to preserve *STAT1* expression, whereas EVs from the probiotic EcN had no effect (Figure 5A). RV infection did not induce significant changes in the mRNA levels of *IRF3* or *ISG15* compared with control cells. The lack of *ISG15* induction was consistent with the reduced expression of *STAT1* and *STAT2* observed in cells infected with the RV SA11 strain. In accordance with the compensatory effect on *STAT1* expression, cells incubated with EcoR12-derived EVs exhibited increased *ISG15* mRNA levels at 24 hpi relative to the other treatment groups (Figure 5A). Collectively, these findings indicated that downregulation of *RIG-I, STAT1,* and *STAT2* expression may be part of the IFN response evasion mechanism employed by the RV SA11 in differentiated Caco-2 cells. These effects were not mitigated by EVs from the probiotic strain EcN. However, EVs derived from the commensal EcoR12 may counteract the inhibitory effects of RV by preserving *STAT1* expression and promoting *ISG15* induction.

Inflammation serves as a protective response to eliminate pathogens and restore homeostasis. IL-8 is a key chemokine in the acute inflammatory response that recruits neutrophils and other immune cells to the infection site. *IL8* expression was assessed by RT-qPCR and ELISA in RV-infected Caco-2 cells at 24 hpi. Infected cells showed increased *IL8* mRNA levels compared with controls. Pretreatment with EVs from the probiotic EcN further enhanced *IL8* expression relative to infected control cells, whereas EVs from the commensal strain EcoR12 had no effect (Figure 5B). Consistently, IL-8 secreted levels at 24 hpi mirrored the transcriptional pattern, confirming higher IL-8 production in cells preincubated with EcN EVs prior to RV infection. Through this mechanism, EcN EVs may enhance the host’s innate immune response and facilitate viral clearance.

### 3.4. EcN and EcoR12 EVs Counteract the RV-Induced Alterations in Epithelial Tight Junction Proteins

Rotavirus infection disrupts epithelial barrier integrity, increasing paracellular permeability [54,55,56]. This effect is associated with altered localization of TJ proteins, whose dynamic assembly is regulated both at the transcriptional and post-translational levels. The RV proteins NSP4 and VP8* activate pathways that promote TJ disassembly and epithelial monolayer disruption, facilitating viral access to cellular receptors located in the basolateral membrane [6,56,57].

**Figure 6:**
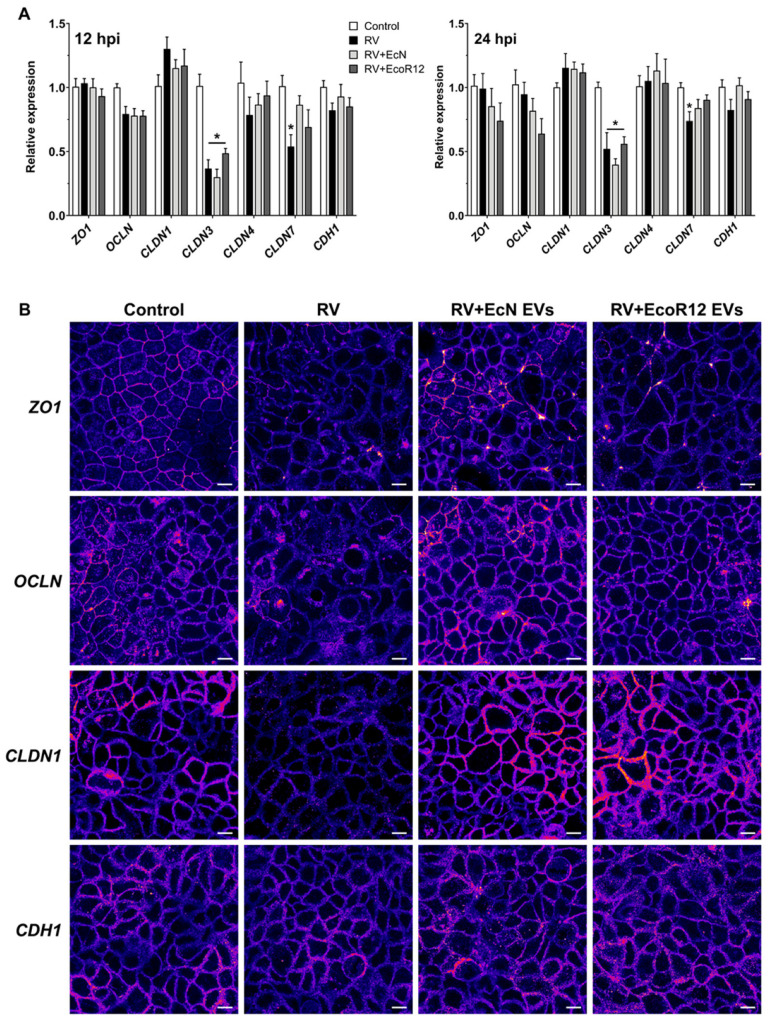
Effect of EcN- and EcoR12-derived EVs on the expression and subcellular distribution of TJ proteins in Caco-2 cell monolayers infected with RV. (**A**) Gene expression analysis in RV-infected cells pretreated with EcN or EcoR12 EVs (5 µg/mL). At 12 hpi and 24 hpi, the relative mRNA levels of the indicated TJ proteins were measured by RT-qPCR using *GAPDH* as the reference gene. Data expressed as mean ± SEM from three biological replicates. Statistical significance: * *p* ≤ 0.05 compared to untreated control cells. (**B**) Immunofluorescence staining of ZO1, occludin (OCLN), claudin-1 (CLDN1) and E-cadherin (CDH1) in RV-infected cells pretreated with EVs (50 µg/mL). Images shown are representative of three independent biological experiments and are coded with Fire look-up table. Scale bar, 20 μm.

First, we analyzed TJ gene expression in RV-infected Caco-2 cells (12 and 24 hpi) and examined whether pretreatment with EcN- or EcoR12-derived EVs (5 µg/mL) could mitigate RV-induced dysregulation. This study included genes encoding the TJ proteins ZO-1, occludin (OCLN), claudin-1 (CLDN1), claudin-3 (CLDN3), claudin-4 (CLDN4), claudin-7 (CLDN7), and E-cadherin (CDH1) (Figure 6A). RV infection did not significantly affect ZO1, OCLN, CLDN1, CLDN4, or CDH1 expression but reduced CLDN3 and CLDN7 mRNA levels at both time points. Pretreatment with EVs failed to prevent CLDN3 downregulation but tended to preserve CLDN7 expression at levels closer to controls, without reaching statistical significance.

The impact of EVs on TJ structure was also evaluated by immunofluorescent labeling of TJ proteins and visualization by confocal microscopy. This approach allows assessment of both protein expression and subcellular localization. Such analysis is critical in the context of RV infection, as this virus activates several signaling pathways that induce post-translational modifications of proteins such as ZO-1, claudin-1, and occludin, leading to their delocalization to intracellular compartments and, consequently, to TJ disruption [6,57]. For this analysis, we used the RV infection model (MOI 4, 1 h) with or without prior treatment with EVs (50 μg/mL) from EcN or EcoR12. The EV dose was selected based on previous studies by our group on the regulation of TJ protein distribution by EcN-derived EVs [43,58]. At 12 hpi, cells were fixed and processed for immunodetection of ZO-1, occludin, claudin-1, and E-cadherin by confocal fluorescence microscopy (Figure 6B). Consistent with previous reports, RV infection markedly reduced the fluorescent signal of ZO-1, occludin, and claudin-1 at intercellular borders, indicating their delocalization to the cytosol and disruption of epithelial junctions. In contrast, E-cadherin distribution was not affected. Pretreatment with EVs from both strains prevented RV-induced alterations in the subcellular localization of occludin and claudin-1, whereas preservation of ZO-1 at tight junctions was observed only with EVs from the probiotic EcN (Figure 6B). Taken together, these results indicate that EcN- and EcoR12-derived EVs exert a protective effect against RV-induced disruption of the epithelial barrier, primarily by preserving the proper distribution and localization of junctional proteins. Through this mechanism, EVs from both strains may hinder RV access to their coreceptors in basolateral membrane and limit their spread within the host tissues.

Beyond their role in reinforcing epithelial cohesion, specific TJ proteins regulate barrier permeability by forming selective paracellular channels for ions and small solutes. Claudin-2 establishes cation-selective pores that facilitate ion exchange and water flux [59]. Its overexpression is linked to increased intestinal permeability in leaky gut syndrome and inflammatory bowel disease [60]. Pathogenic viruses and enteric bacteria exploit this mechanism by inducing claudin-2 to disrupt barrier integrity [61]. Conversely, downregulation of claudin-2 (encoded by gene *CLDN2*) contributes to probiotic-mediated barrier protection. In this context, we previously reported that EcN EVs reduce *CLDN2* expression in intestinal epithelial models [43].

**Figure 7:**
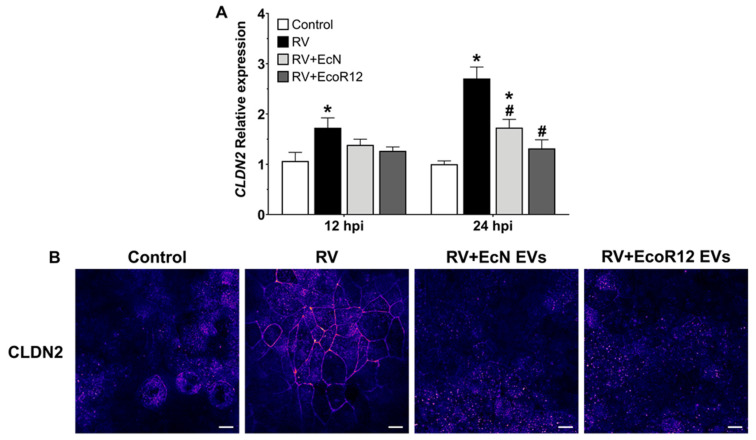
Effect of EcN- and EcoR12-derived EVs on the expression and subcellular distribution of claudin-2 (CLDN2) in Caco-2 cell monolayers infected with RV. (**A**) Gene expression analysis in RV-infected cells pretreated with EcN or EcoR12 EVs (5 µg/mL). At 12 hpi and 24 hpi, the relative *CLDN2* mRNA levels were measured by RT-qPCR using *GAPDH* as the reference gene. Data expressed as mean ± SEM from three biological replicates. Statistical significance: * *p* ≤ 0.05 compared to untreated control cells; # *p* ≤ 0.05 compared to RV-infected cells. (**B**) Immunofluorescence staining of claudin-2 in RV-infected cells pretreated with EVs (50 µg/mL). Images shown are representative of three independent biological experiments and are coded with Fire look-up table. Scale bar, 20 μm.

Given the link between claudin-2 overexpression and increased intestinal permeability during enteric infection, we investigated claudin-2 expression and subcellular localization in RV-infected Caco-2 cells, and evaluated whether EcN and EcoR12 EVs could prevent the RV-induced alterations. RV infection markedly upregulated *CLDN2* mRNA levels, an effect significantly attenuated by EVs from both strains (Figure 7A). Confocal immunofluorescence confirmed increased claudin-2 abundance and its predominant localization at the plasma membrane in RV-infected cells relative to controls and EV-treated cells (Figure 7B). These findings suggest that EVs from both strains mitigate RV-induced barrier disruption through complementary mechanisms: preserving TJs’ integrity via post-translational regulation of occludin, claudin-1, and ZO-1, and suppressing claudin-2 expression, thereby potentially reducing paracellular water efflux and limiting RV-associated diarrhea.

### 3.5. EcN and EcoR12 EVs Interfere with Mucin Production and Secretion in RV-Infected Cells

Several studies using cellular and preclinical models have reported an increase in mucin expression and secretion during the course of rotavirus infection [7,10,11]. Mucin secretion has been proposed as a protective host response that entraps and eliminates the virus through fecal excretion. However, excessive mucin production may exacerbate diarrhea and promote viral transmission among individuals [8]. In this context, we investigated the effect of EcN- and EcoR12-derived EVs on mucin expression and secretion in RV-infected goblet cells. The LS174T cell line was employed as a goblet cell model, as it is widely recognized as a robust system for studying mucin-2 (MUC2) expression [62].

**Figure 8:**
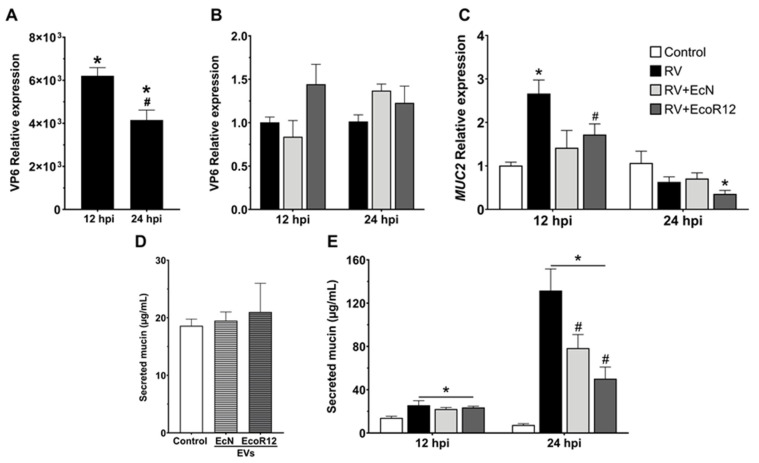
Effects of EcN- and EcoR12-derived EVs on RV infection and mucin production in goblet cell-like LS174T cells. Cells were infected with RV strain SA11 (MOI 4) for 1 h (time 0). When indicated, cells were pretreated with EcN or Ecor12 EVs (5 µg/mL) prior to infection. (**A**) VP6 RNA levels were quantified by RT-qPCR and normalized to GAPDH expression. Results are expressed as fold change relative to VP6 RNA levels at time 0. (**B**) VP6 RNA levels measured by RT-qPCR in RV-infected LS174T cells pretreated with EVs of the indicated strains. Data are expressed as fold-change relative to RV-infected cells (set at 1). (**C**) *MUC2* RNA levels measured by RT-qPCR in RV-infected LS174T cells pretreated with EVs of the indicated strains. Data are expressed as fold-change relative to non-infected control cells (set at 1). (**D**) Effect of EcN- and EcoR12-derived EVs on secreted mucin levels measured in culture supernatants of non-infected LS174T cells using Alcian blue staining. (**E**) Quantification of secreted mucin by RV-infected cells pretreated with EVs of the indicated strains. Statistical significance: * *p* ≤ 0.05 vs. control cells; # *p* ≤ 0.05 vs. RV-infected cells.

First, the susceptibility of this cell type to infection by the RV SA11 strain was evaluated. Since the LS174T cell line does not form a differentiated monolayer, cells were cultured until reaching 80–90% confluence and then infected with RV (MOI 4) for 1 h. After washing with serum-free medium to remove non-internalized virus (0 hpi), cells were incubated for 12 and 24 h, and viral load was quantified by RT-qPCR using VP6-specific primers (Figure 8A). The relative *VP6* RNA levels in LS174T cells were markedly lower than those observed in Caco-2 cells used as an enterocyte model (Figure 1). Moreover, *VP6* RNA abundance decreased over time, being significantly lower at 24 than at 12 hpi. The reduced viral load in goblet cells is consistent with the fact that RV primarily infects and replicates in mature enterocytes of the intestinal villus epithelium [3]. Importantly, pretreatment with EcN- and EcoR12-derived EVs (5 µg/mL) did not alter viral RNA levels (Figure 8B).

These results indicated that EVs derived from these probiotic and commensal *E. coli* strains do not prevent RV entry or infection in goblet cells, although they may interfere with virus-induced mucin secretion. To address this hypothesis, *MUC2* expression was analyzed in LS174T cells infected with RV SA11, with or without prior EV treatment. At the mRNA level, *MUC2* expression increased at 12 hpi in RV-infected cells, and this effect was partially counteracted by EVs from both strains (Figure 8C). By 24 hpi, *MUC2* expression in infected cells returned to control levels, with a lower expression observed in cells treated with EVs from the commensal EcoR12 strain. To quantify mucin secreted levels, supernatants from RV-infected LS174T cells, with or without EV pretreatment (5 μg/mL), were collected at 12 and 24 hpi and processed for mucin release assays as described in the Methods Section 2.11. As a control, mucin secretion was also quantified in LS174T cells treated with EcN or EcoR12 EVs in the absence of RV. Under non-infectious conditions, EVs from both strains did not affect the basal mucin secretion of goblet cells (Figure 8D). In contrast, RV infection induced a marked increase in extracellular mucin concentration compared with control cells. Mucin secretion doubled at 12 hpi and increased nearly tenfold at 24 hpi relative to uninfected cells (Figure 8E). Pretreatment with EcN or EcoR12 EVs had no apparent effect at 12 hpi but significantly reduced mucin secretion at 24 hpi, with a stronger effect observed for EcoR12 EVs. Overall, these findings indicated that EVs from these intestinal *E. coli* strains do not inhibit rotavirus infection in goblet cells but attenuate virus-induced mucin production and secretion. Through this mechanism, EVs derived from beneficial strains of the gut microbiota may reduce viral dissemination and limit the transmission of infection.

## 4. Discussion

Rotavirus remains a leading cause of severe diarrhea and child mortality worldwide, with current vaccines showing reduced efficacy in low-income regions and no specific antiviral treatment available [63,64,65]. Evidence increasingly highlights the gut microbiota as a key modulator of host–pathogen interactions, prompting interest in probiotics as preventive and therapeutic strategies [23,24,25,26,27,28]. Particularly, the probiotic EcN has demonstrated protective effects against rotavirus in preclinical models by inhibiting viral attachment, enhancing antiviral immune responses, and strengthening epithelial barrier function [24,25,66,67]. Clinical studies further support the benefits of certain probiotics, such as *Lactobacillus rhamnosus* GG, *L. reuteri*, and *Saccharomyces boulardii*, in reducing the duration and severity of rotavirus diarrhea, although efficacy varies with host factors [68,69,70]. Safety concerns regarding live bacteria have driven interest in postbiotics, non-viable microbial derivatives, as potentially safer and more consistent alternatives for managing enteric infections [71,72,73].

Probiotic-derived EVs have emerged as promising postbiotic candidates due to their role in intercellular communication, ability to cross epithelial barriers, and capacity to deliver bioactive molecules to host cells [32,74,75]. While most studies have focused on inflammatory, metabolic, or neuroimmune disorders, evidence in neonatal viral infections is scarce. Previously, we showed that EVs from EcN and EcoR12 mitigated diarrhea and enhanced antiviral humoral and cellular immunity in suckling rats infected with RV [38]. Nonetheless, the molecular mechanisms induced by EcN- or EcoR12-derived EVs in enterocytes, the primary site of infection, remain largely unknown. Here, using polarized Caco-2 cells as a model of mature enterocytes, we demonstrate that these EVs exert protective effects at multiple stages of the RV infection cycle. Some mechanisms are shared by EVs from both *E. coli* strains, while others are strain-specific. Although Cryo-TEM analysis confirmed the vesicular identity and high purity of the EV preparations [41], we cannot completely exclude the possibility that minor co-isolated non-vesicular components may contribute to the observed effects.

In the established infection model, treatment with EcN- or EcoR12-derived EVs prior to infection resulted in reduced VP6 RNA and protein levels, an effect evident as early as 3 hpi. This finding suggested that EVs from both strains could interfere with RV replication by acting at early stages of the infection process, including virus binding and entry. The increase in the intracellular Ca^2+^ concentration, the formation of ROS, and the activation of COX-2 are essential for RV replication and assembly, and their activation by RV was counteracted by the EVs from both EcN and EcoR12 strains.

Elevated cytosolic Ca^2+^ is required for RV replication-related processes, including autophagy, viroplasm formation, and capsid assembly [3]. The viral protein responsible for increasing intracellular Ca^2+^ levels in infected cells is the viroporin NSP4, which inserts into the endoplasmic reticulum membrane and promotes Ca^2+^ release into the cytosol. The resulting depletion of Ca^2+^ stores activates the stromal interaction molecule STIM1, triggering extracellular Ca^2+^ influx through calcium channels [4]. In addition, RV-infected cells release ADP, which elevates Ca^2+^ in neighboring non-infected cells, facilitating viral spread [76,77]. The ability of EcN and EcoR12 EVs to reduce the RV-induced intracellular increase in intracellular Ca^2+^ was consistent with the observed inhibition of RV infection, suggesting that EVs interfere with Ca^2+^-dependent steps of the viral cycle. Moreover, by preserving Ca^2+^ homeostasis, EVs may also mitigate additional RV-associated epithelial cell dysfunction, including diarrhea and disruption of cytoskeletal and barrier integrity [42,76].

High levels of ROS are a common feature of many viral infections. Viruses can induce ROS production through multiple mechanisms, including ER stress–mediated calcium release, mitochondrial dysfunction, and interference with host antioxidant defenses [51]. Among other viruses, RV also exploits ROS to promote replication and pathogenesis [5]. Accordingly, antioxidant treatments have been shown to reduce RV infection and diarrhea in vitro and in patients [78]. The protective effect of certain probiotics against NSP4-dependent chloride secretion has also been linked to their antioxidant activity [44,79]. Our findings demonstrate that EVs from the beneficial *E. coli* strains EcN and EcoR12 possess antioxidant activity, significantly reducing intracellular ROS levels in RV-infected Caco-2 cells during early infection. At 30 min post-infection, ROS levels in EV-pretreated cells were reduced to less than 50% of those in untreated RV-infected controls. This effect is consistent with our previous observation that EcN and EcoR12 EVs upregulate antioxidant response genes, including *SOD* and *CAT*, in the same cell line [80]. Thus, in EV-pretreated cells, the enhancement of cellular antioxidant defenses likely contributes to the suppression of early RV-induced ROS and may interfere with subsequent viral replication.

PGE_2_ is an eicosanoid mediator produced by cyclooxygenases (COX-1 and the inducible COX-2 isoform) with key roles in inflammation and immune regulation. PGE_2_ also modulates viral infections by helping viral attachment, replication, and protein expression in a virus- and cell-type–dependent manner [47,48]. RV infection upregulates COX-2 expression in epithelial cells and animal models, and elevated PGE_2_ levels have been detected in stool samples from RV-infected children. Remarkably, COX-2 inhibition with acetylsalicylic acid reduces diarrhea duration and viral load [48,81]. Our results confirmed that RV infection induces *COX2* expression in Caco-2 monolayers, whereas treatment with EVs from EcN or EcoR12 prevents this induction. The reduction in *COX2* mRNA levels in EV-treated cells is likely to limit PGE_2_ overproduction, resulting in decreased viral replication, as reflected by reduced VP6 RNA and protein levels. This fact is consistent with the reported effects of COX-2 inhibitors. Additionally, by this mechanism, EVs from these microbiota-derived *E. coli* strains may help preserve Th1-mediated antiviral responses, which are typically suppressed by PGE_2_. Notably, EcN-derived EVs have also been shown to inhibit *COX2* overexpression in an in vitro IL-1β–induced inflammation model and in a murine colitis model [80,82]. Additional functional experiments using Ca^2+^ chelators, ROS scavengers, and COX-2 inhibitors are required to dissect the main molecular pathways regulated by EVs and clarify their mechanistic roles.

Innate immunity is rapidly mobilized upon viral exposure to suppress viral replication and, ideally, eliminate the pathogen before the adaptive immune response is engaged. This response is initiated through host recognition of viral components, leading to the induction of chemokines, pro-inflammatory mediators, and type I IFNs, which together establish a robust antiviral state. However, many viruses have evolved strategies to counteract or evade these innate defenses, enabling more efficient replication and spread [53]. Our results provide evidence that EVs from EcN and EcoR12 can interfere with RV-induced immune evasion mechanisms in epithelial cells, enhancing type I interferon-based innate immunity and neutrophil recruitment, with effects displaying clear strain specificity.

First, EVs from the probiotic EcN enhance the innate immune response in RV-infected epithelial cells by increasing IL-8 production, thereby improving the recruitment of neutrophils and monocytes to aid pathogen clearance. This effect is strain-specific, as EVs from EcoR12 did not alter IL-8 levels in RV-infected cells. Although EcN EVs have anti-inflammatory activity in other inflammatory conditions, such as IBD [80,82], in the RV infection model, the increase in IL-8 likely represents a beneficial alarm signal that supports early antiviral defense involving cellular immunity. These findings underscore the context-dependent immunomodulatory capacity of microbiota-derived EVs, enhancing protective inflammation during infection while dampening pathological inflammation in disease settings. Concerning the effects on the type I IFN response, infection of Caco-2 cells by RV SA11 downregulated the expression of key genes of this pathway, including *RIG-I*, *STAT1*, and *STAT2*, thereby impairing ISG activation and antiviral defense. While EVs from the tested strains did not restore *RIG-I* or *STAT2* expression, EVs from the commensal EcoR12 effectively prevented RV-induced *STAT1* downregulation and enhanced *ISG15* expression in infected cells. This suggests that EcoR12 EVs can partially counteract RV immune evasion and help maintain type I IFN-mediated antiviral activity. Further research is required to assess whether these EVs also impact post-translational mechanisms controlling IFN signaling.

Among the benefits of EcN and EcoR12-derived EVs is their ability to interfere with rotavirus dissemination by modulating proteins that maintain the intestinal epithelial barrier. Both the mucin layer that covers the intestinal epithelium and the formation of TJs between enterocytes constitute physical barriers that prevent RV infection. Indeed, viruses have evolved mechanisms to disrupt these structures, facilitating access to enterocytes and dissemination to other host tissues [8,83]. The results of this study indicated that EVs derived from the probiotic EcN and the commensal EcoR12 regulate *MUC2* expression and secretion in goblet cells, as well as the subcellular localization of proteins involved in TJ assembly in intestinal epithelial cells, counteracting RV-induced alterations.

RV infection increases the expression and secretion of MUC2, the main mucin produced by intestinal goblet cells, likely as a host response to trap the virus. While enhanced MUC2 secretion can restrict RV access to the epithelial surface, it may also facilitate viral dissemination via feces. Our results confirmed that RV increases *MUC2* expression during early infection in goblet cells. EVs from EcN and EcoR12 prevented RV-induced *MUC2* overexpression and reduced secreted MUC2 levels without affecting viral entry or replication. These findings are consistent with previous in vivo evidence showing that EVs from these strains prevent RV-induced mucin depletion in neonatal rats [38]. Because EVs did not alter VP6 RNA levels in LS174T cells, the reduction in mucin secretion observed at later stages is more consistent with indirect EV-mediated modulation of host signaling pathways regulating mucin production or secretion, rather than with a direct antiviral effect in goblet cells. Thus, EVs from EcN and EcoR12 may limit RV transmission by inhibiting virus-driven mucin overproduction and release.

TJs between intestinal epithelial cells form the primary barrier restricting the entry of harmful agents and pathogens and maintain apical–basolateral compartmentalization in polarized epithelia. However, some TJ proteins can serve as entry points for pathogens [83]. Several viruses, including RV, can overcome this barrier by using TJ proteins as receptors or by disrupting TJ integrity. RV uses various integrins as cellular receptors, which are normally restricted to the basolateral membrane beneath TJs. The RV capsid protein VP4, cleaved by trypsin into VP8* and VP5*, contributes to TJ disruption, allowing RV access to these coreceptors [6,57]. RV-induced TJ remodeling occurs mainly through post-translational mechanisms, leading to the mislocalization of ZO-1, claudin-1, and occludin without altering their mRNA expression [6,55,56,57]. In agreement with this information, RV infection in our model reduced *CLDN3* and *CLDN7* expression but did not affect *CLDN1*, *CLDN4*, *OCLN*, *ZO1*, or *CDH1* mRNA levels. EVs from EcN and EcoR12 tended to preserve *CLDN7* expression. Regarding subcellular distribution of TJ proteins, RV caused mislocalization of claudin-1, occludin, and ZO-1. Importantly, EVs from both EcN and EcoR12 prevented delocalization of claudin-1 and occludin, and specifically, EcN EVs also maintained ZO-1 localization at the cell boundaries. These results suggest that EVs interfere with RV-induced post-translational modifications of TJ proteins, thereby preserving epithelial barrier integrity and limiting viral entry and spread. The reduced viral load observed in EV-treated cells may partly result from this protective effect. TJs also regulate intestinal permeability. In this context, RV proteins such as NSP4, VP5* and VP8* contribute to diarrhea by increasing paracellular water flow [84]. Moreover, RV upregulates claudin-2, a leaky protein associated with increased permeability [61,85]. In our model, RV increased *CLDN2* expression and its localization at apical TJs, whereas EVs from EcN and EcoR12 prevented these changes. Thus, EVs from both strains may counteract RV-induced barrier disruption by stabilizing TJ structure and preventing claudin-2 mediated water loss.

This study provides the first evidence that EVs from EcN and EcoR12 protect against rotavirus infection through multiple, strain-specific mechanisms. Understanding these pathways will enhance knowledge of how the microbiota interacts with the host during viral infections and may pave the way for effective and safe postbiotic interventions against viral gastroenteritis.

## 5. Conclusions

This study demonstrates that EVs derived from the probiotic EcN and the commensal EcoR12 exert multifaceted protective effects against RV pathology in a mature enterocyte model, with several actions being strain-specific. EVs from both strains disrupt early steps of RV replication by counteracting key host processes required for viral multiplication, including Ca^2+^ mobilization, ROS generation, and COX-2–mediated PGE_2_ synthesis. Moreover, they reduce virus-induced MUC2 production and secretion, a mechanism that may limit viral shedding and transmission. Both types of EVs restore RV-altered tight junction organization, thereby restricting viral access to basolateral coreceptors. Moreover, EVs enhance innate antiviral defenses, although through distinct, strain-dependent pathways. EcN EVs boost IL-8-mediated cellular responses, whereas EcoR12 EVs preserve IFN-related signaling by preserving *STAT1* expression and increasing *ISG15.*

Overall, these findings provide strong evidence that EVs from EcN and EcoR12 act at multiple cellular levels to limit RV replication, spread, and immune evasion, supporting their potential as safe and effective postbiotic candidates for preventing or treating rotavirus infection.

## Data Availability

The original contributions presented in this study are included in the article/Supplementary Material. Further inquiries can be directed to the corresponding authors.

## Supplementary Materials

The following supporting information can be downloaded at https://www.mdpi.com/article/10.3390/pharmaceutics18010120/s1. Table S1: Sequences of primers used for quantitative RT-PCR. Refs. [80,86,87,88,89,90,91,92,93,94,95,96,97,98] cited in Supplementary Materials.

## Abbreviations

The following abbreviations are used in this manuscript:

CLDN	Claudin
COX	Cyclooxygenase
DCFDA	2′,7′-dichlorofluorescin diacetate
DMEM	Dulbecco’s Modified Eagle Medium
EcN	*Escherichia coli* Nissle 1917
ELISA	Enzyme-linked immunosorbent assay
EVs	Extracellular vesicles
FBS	Fetal bovine serum
GAPDH	Glyceraldehyde-3-phosphate dehydrogenase
IFN	Interferon
IL	Interleukin
ISG	Interferon-stimulated genes
LDH	Lactate dehydrogenase
MOI	Multiplicity of infection
MUC2	Mucin 2
NSP	Nonstructural protein
NF-kB	Nuclear factor kappa B
OCLN	Occludin
PFU	Plaque-forming units
PGE2	Prostaglandin E2
RIG-I	Retinoic acid-inducible gene 1
ROS	Reactive oxygen species
RT-qPCR	Quantitative reverse transcription-polymerase chain reaction
TCR	T cell receptor
TJ	Tight junctions
TLR	Toll-like receptor
SEM	Standard error of the mean
STAT	Signal transducer and activator of transcription
VP	Viral protein
ZO-1	Zonula occludens 1
