# Supplementary material for: Extracellular Vesicles from Probiotic and Beneficial Escherichia coli Strains Exert Multifaceted Protective Effects Against Rotavirus Infection in Intestinal Epithelial Cells
Source: Pharmaceutics. 2026 Jan 18;18(1):120. doi: 10.3390/pharmaceutics18010120 (PMC12844683; doi:10.3390/pharmaceutics18010120)
Supplement: Supplementary file 1 [file pharmaceutics-18-00120-s001.zip › pharmaceutics-4067564-supplementary.pdf]

Table S1. Sequences of primers used for quantitative RT-PCR.

GENE	**Forward Sequence**	**Reverse Sequence**	**References**
CLDN1	GCCCCAGTGGAGGATTTACT	GTTTTGGATAGGGCCTTGGT	[1]
CLDN2	ACCTGCTACCGCCACTCTGT	CTCCCTGGCCTGCATTATCTC	[2]
CLDN3	GAGATGGGAGCTGGGTTGTA	GGATCTTGGTGGGTGCATAC	[3]
CLDN4	CAGCGCGATGCCCATTA	CGCATCAGGACTGGCTTTATCTC	[4]
CLDN7	AATTTTCATCGTGGCAGGTC	AGGACAGGAACAGGAGAGAGCA	[5]
COX2	CAGCACTTCACGCATCAGTT	CGCAGTTTACGCTGTCTAGC	*[6]*
CDH1	GAAGGTGACAGAGCCTCTGGAT	GATCGGTTACCGTGATCAAAAT	*[7]*
GAPDH	GTCAACGGATTTGGTCGTATTG	TGGAAGATGGTGATGGGATTT	[8]
IL8	CTGGCCGTGGCTCTCTTG	GGGTGGAAAGGTTTGGAGTATG	[1]
IRF3	ACCAGCCGTGGACCAAGAG	TACCAAGGCCCTGAGGCAC	[9]
ISG15	CTCTGAGCATCCTGGTGAGGAA	AAGGTCAGCCAGAACAGGTCGT	[10]
MUC2	CACCTGTGCCCTGGAAGGC	CGGTCACGTGGGGCAGGTTC	[2]
OCLN	TCCTATAAATCCACGCCGGTTC	CTCAAAGTTACCACCGCCTGCTG	[11]
RIG1	GACCCTGGACCCTACCTACA	CCAACTTTCAATGGCTTCAT	[12]
STAT1	ATGGCAGTCTGGCGGCTGAATT	CCAAACCAGGCTGGCACAATTG	[13]
STAT2	CAGGTCACAGAGTTGCTACAGC	CGGTGAACTTGCTGCCAGTCTT	[14]
VP6	CAGTGATTCTCAGGCCGAATA	GGCGAGTACAGACTCACAAA	[8]
ZO1	CGGGACTGTTGGTATTGGCTAGA	GGCCAGGGCCATAGTAAAGTTTG	[11]

References

1. Olivo-Martínez, Y.; Martínez-Ruiz, S.; Cordero-Alday, C.; Bosch, M.; Badia, J.; Baldoma, L. Modulation of Serotonin-Related Genes by Extracellular Vesicles of the Probiotic Escherichia Coli Nissle 1917 in the Interleukin-1β-Induced Inflammation Model of Intestinal Epithelial Cells. *Int J Mol Sci* 2024, *25*, doi:10.3390/ijms25105338.

2. Olivo-Martínez, Y.; Bosch, M.; Badia, J.; Baldomà, L. Modulation of the Intestinal Barrier Integrity and Repair by Microbiota Extracellular Vesicles through the Differential Regulation of Trefoil Factor 3 in LS174T Goblet Cells. *Nutrients* 2023, *15*, 2437, doi:10.3390/nu15112437.

3. Shin, S.-B.; Kim, Y.-M.; Park, H.-Y. Pinoresinol Enhances Oral Barrier Integrity and Function in Human Buccal Cell Monolayers. *PLoS One* 2025, *20*, e0331242, doi:10.1371/journal.pone.0331242.

4. Kaarteenaho, R.; Merikallio, H.; Lehtonen, S.; Harju, T.; Soini, Y. Divergent Expression of Claudin -1, -3, -4, -5 and -7 in Developing Human Lung. *Respir Res* 2010, *11*, 59, doi:10.1186/1465-9921-11-59.

5. Tokuhara, Y.; Morinishi, T.; Matsunaga, T.; Sakai, M.; Sakai, T.; Ohsaki, H.; Kadota, K.; Kushida, Y.; Haba, R.; Hirakawa, E. Nuclear Expression of Claudin-3 in Human Colorectal Adenocarcinoma Cell Lines and Tissues. *Oncol Lett* 2017, doi:10.3892/ol.2017.7281.

6. Wang, Z.; Hao, J.; Chen, D. Long Noncoding RNA Nuclear Enriched Abundant Transcript 1 (NEAT1) Regulates Proliferation, Apoptosis, and Inflammation of Chondrocytes via the MiR-181a/Glycerol-3-Phosphate Dehydrogenase 1-Like (GPD1L) Axis. *Medical Science Monitor* 2019, *25*, 8084–8094, doi:10.12659/MSM.918416.

7. Osman, I.O.; Garrec, C.; de Souza, G.A.P.; Zarubica, A.; Belhaouari, D.B.; Baudoin, J.-P.; Lepidi, H.; Mege, J.-L.; Malissen, B.; Scola, B. La; et al. Control of CDH1/E-Cadherin Gene Expression and Release of a Soluble Form of E-Cadherin in SARS-CoV-2 Infected Caco-2 Intestinal Cells: Physiopathological Consequences for the Intestinal Forms of COVID-19. *Front Cell Infect Microbiol* 2022, *12*, doi:10.3389/fcimb.2022.798767.

8. Sarkar, R.; Nandi, S.; Lo, M.; Gope, A.; Chawla-Sarkar, M. Viperin, an IFN-Stimulated Protein, Delays Rotavirus Release by Inhibiting Non-Structural Protein 4 (NSP4)-Induced Intrinsic Apoptosis. *Viruses* 2021, *13*, 1324, doi:10.3390/v13071324.

9. Hu, J.; Lou, D.; Carow, B.; Winerdal, M.E.; Rottenberg, M.; Wikström, A.-C.; Norstedt, G.; Winqvist, O. LPS Regulates SOCS2 Transcription in a Type I Interferon Dependent Autocrine-Paracrine Loop. *PLoS One* 2012, *7*, e30166, doi:10.1371/journal.pone.0030166.

10. Li, T.; Liu, H.; Jiang, N.; Wang, Y.; Wang, Y.; Zhang, J.; Shen, Y.; Cao, J. Comparative Proteomics Reveals Cryptosporidium Parvum Manipulation of the Host Cell Molecular Expression and Immune Response. *PLoS Negl Trop Dis* 2021, *15*, e0009949, doi:10.1371/journal.pntd.0009949.

11. Toschi, A.; Rossi, B.; Tugnoli, B.; Piva, A.; Grilli, E. Nature-Identical Compounds and Organic Acids Ameliorate and Prevent the Damages Induced by an Inflammatory Challenge in Caco-2 Cell Culture. *Molecules* 2020, *25*, 4296, doi:10.3390/molecules25184296.

12. He, J.; Kuang, Y.; Xu, K.; Huang, R.; Yang, X.; Deng, L.; Feng, X.; Ren, Y.; Yang, J.; Yuan, L. TRIM38 Inhibits Zika Virus by Upregulating RIG-I/MDA5 Pathway and Promoting Ubiquitin-Mediated Degradation of Viral NS3 Protein. *Viruses* 2025, *17*, 199, doi:10.3390/v17020199.

13. Li, D.; Zhang, J.; Yang, W.; Li, P.; Ru, Y.; Kang, W.; Li, L.; Ran, Y.; Zheng, H. African Swine Fever Virus Protein MGF-505-7R Promotes Virulence and Pathogenesis by Inhibiting JAK1- and JAK2-Mediated Signaling. *Journal of Biological Chemistry* 2021, *297*, 101190, doi:10.1016/j.jbc.2021.101190.

14. Yoshikawa, R.; Sakurai, Y.; Kondo, S.; Kimura, M.; Yasuda, J. Crimean-Congo Hemorrhagic Fever Virus NSm Protein Inhibits the Type I Interferon Signaling by Binding to STAT2. *PLoS Negl Trop Dis* 2025, *19*, e0013695, doi:10.1371/journal.pntd.0013695.
